# Regional Variation in Acute Kidney Injury Requiring Dialysis in the English National Health Service from 2000 to 2015 – A National Epidemiological Study

**DOI:** 10.1371/journal.pone.0162856

**Published:** 2016-10-17

**Authors:** Nitin V. Kolhe, Richard J. Fluck, Andrew W. Muirhead, Maarten W. Taal

**Affiliations:** 1 Department of Renal Medicine, Royal Derby Hospital, Uttoxeter Road, Derby, DE22 3NE, United Kingdom; 2 Department of Public Health, Derby City Council, Corporation Street, Derby, DE1 2FS, United Kingdom; 3 Division of Medical Sciences and Graduate Entry Medicine, University of Nottingham, Uttoxeter Road, Derby, DE22 3NE, United Kingdom; Postgraduate Medical Institute, INDIA

## Abstract

**Background:**

The absence of effective interventions in presence of increasing national incidence and case-fatality in acute kidney injury requiring dialysis (AKI-D) warrants a study of regional variation to explore any potential for improvement. We therefore studied regional variation in the epidemiology of AKI-D in English National Health Service over a period of 15 years.

**Method:**

We analysed Hospital Episode Statistics data for all patients with a diagnosis of AKI-D, using ICD-10-CM codes, in English regions between 2000 and 2015 to study temporal changes in regional incidence and case-fatality.

**Results:**

Of 203,758,879 completed discharges between 1^st^ April 2000 and 31^st^ March 2015, we identified 54,252 patients who had AKI-D in the nine regions of England. The population incidence of AKI-D increased variably in all regions over 15 years; however, the regional variation decreased from 3·3-fold to 1·3-fold (p<0·01). In a multivariable adjusted model, using London as the reference, in the period of 2000–2005, the North East (odd ratio (OR) 1·38; 95%CI 1·01, 1·90), East Midlands (OR 1·38; 95%CI 1·01, 1·90) and West Midlands (OR 1·38; 95%CI 1·01, 1·90) had higher odds for death, while East of England had lower odds for death (OR 0·66; 95% CI 0·49, 0·90). The North East had higher OR in all three five-year periods as compared to the other eight regions. Adjusted case-fatality showed significant variability with temporary improvement in some regions but overall there was no significant improvement in any region over 15 years.

**Conclusions:**

We observed considerable regional variation in the epidemiology of AKI-D that was not entirely attributable to variations in demographic or other identifiable clinical factors. These observations make a compelling case for further research to elucidate the reasons and identify interventions to reduce the incidence and case-fatality in all regions.

## Introduction

Acute kidney injury requiring dialysis (AKI-D) has increased considerably over the last 15 years[1]. The national rise in incidence of acute kidney injury (AKI) has several ramifications in terms of cost to the health services resulting not only from cost of therapy but also from the later consequences of AKI including development of chronic kidney disease and cardiovascular disease [2–4]. Though mortality in less severe forms of AKI has decreased over last 15 years, this is not case for the most severe form of AKI, which has remained unchanged in the last decade in England[1, 5]. In recent years, it has become clear that even the national incidence and case-fatality of AKI may be subject to regional variation [6]. Understanding the reasons for this variation may be a key step in developing strategies to achieve improvement. In other areas of healthcare, principally surgery, it has been reported that unwarranted variation is due to factors other than patient characteristics including access to care, socioeconomic factors, provider capacity, medical malpractice pressure, and different local practices [7–11]. Despite the public health burden of AKI-D in England, it is unclear if regional variation exists in AKI-D despite the existence of a single National Health System (NHS).

To address this gap in knowledge, we combined a national database of hospital admissions and discharge with census data from office of national statistics over a period of 15 years to determine the variation as well as trends over time in the regional incidence and case fatality of AKI-D in English regions. We also explored determinants of the regional variation in AKI-D.

## Materials and Methods

### Data source

The data for 2000–2015 was extracted from the Hospital Episode Statistics (HES), a data warehouse containing details of all admissions at NHS hospitals in England. As described previously, we extracted HES data as a finished discharge spell, which is defined as the total continuous completed in-hospital stay of a patient on premises controlled by a health-care provider during which medical care is the responsibility of one or more doctors [1]. To reduce the risk of bias, we excluded patients who did not have a documented discharge outcome in the HES database, as they would be categorized as alive in HES database.

### Definitions

We identified all cases of AKI by using validated International Classification of Diseases, Tenth Revision, Clinical Modification (ICD-10-CM) codes for acute renal failure in any diagnoses codes, in keeping with the objective of the study. As described previously, patients with AKI-D were identified by including procedure code for hemodialysis (X40.3) or hemofiltration (X40.4) in any of the 24 procedures [1]. Patients’ with chronic kidney disease (CKD) starting long-term dialysis (N18.5) and end stage renal disease (N18.6) were excluded. Procedure codes for AVF (L74·2) or AVG (L74·3) during the inpatient admission were also excluded. The algorithm has been shown to be sensitive and specific, with a high positive and negative predictive value (all >90%) [12]. HES data stratifies patient location into 16 different regions. The geographic regions in England were stratified as per the Office of National Statistic (ONS) into nine regions: North East, North West, Yorkshire and Humber, East Midlands, West Midlands, East of England, London, South East and South West. Patients in geographical locations outside these nine regions were excluded. Patients who were not discharged during the study period were excluded. We also obtained all completed hospital discharges from each region to estimate the effect of hospitalization on AKI-D incidence rates, along with number of qualified nephrologists in each region from 2000 to 2015 from Health and Social Care Information Centre in the annual census of medical staff in the NHS.

### Outcome measures

We obtained data on patient demographics, details of hospital admission and discharge, length of stay, comorbidities and operations from the HES database. We obtained mid-year population data for each region in each year from 2000 to 2015 from the ONS to obtain population incidence of AKI-D for each region.

### Validation

The National Confidential Enquiry in Patient Outcomes and Death validated ICD-10 codes for AKI and found 74·1% sensitivity, 96% specificity, 90·9% positive predictive value and 87·2% negative predictive value for identifying AKI in NHS hospitals in England [13]. Similarly, another study performed in England found excellent positive predictive value for ICD-10 N17 codes when compared against the KDIGO criteria for AKI with no suggestion of marked changes in coding of AKI between 2005 and 2010 [14].

### Statistical analysis

All analyses were performed using IBM SPSS Statistics for Windows, Version 22·0. Armonk, NY: IBM Corp. Data during the 15-year period were divided into three five-year periods (April 2000 to March 2005, April 2005 to March 20010 and April 2010 to March 2015) and also presented by individual year. Age was categorized into the groups <65, 65–74, 75–84 and >85 years. Charlson’s comorbidity index (CCI) greater than five were grouped into one category as described previously [15]. Method of admission was defined as one of elective admission; emergency admission; maternity admission; other admission or unknown. Ethnicity was grouped into six categories as White, Mixed, Asian, Black, other ethnic groups and ethnicity not stated/unknown. AKI in diagnosis codes were categorized into ‘AKI in primary diagnosis code’, ‘AKI in secondary diagnosis code’ and ‘AKI in other diagnoses codes’ as per the presence of ICD10 codes of N17. Continuous variables are reported as mean with 95% confidence interval; these were compared with the t-test under the Central Limit Theorem. Categorical variables are presented as proportions and compared with the chi square test. We created a multivariable logistic regression models using hospital discharge status as outcome and region as the predictor for three five-year periods: 2000–05, 2005–10, 2010–15, adjusting for gender, age group, period of admission, AKI in diagnoses codes, admission method, CCI and ethnicity. We used London as the reference region for all our analyses. We also performed multivariable logistic regression for each region, adjusting for the above variables, to assess if adjusted case-fatality improved in a defined region over a period of 15 years. We used the year period 2000–01 as the reference. Patients with missing data were excluded from analysis. The sensitivity of the findings was examined by performing the analysis by excluding unknown ethnicity and also, using CCI as continuous variable. We investigated the difference in the effect size with improvement in ethnicity recording in HES (see appendix 1). The study protocol was deemed exempt from ethical approval because the research involved non-identifiable information, previously collected in the course of normal care and available for public use. The study was conducted according to the principles expressed in the Declaration of Helsinki.

This study is registered with ClinicalTrials.gov, number NCT02675296

## Results

**Fig 1:**
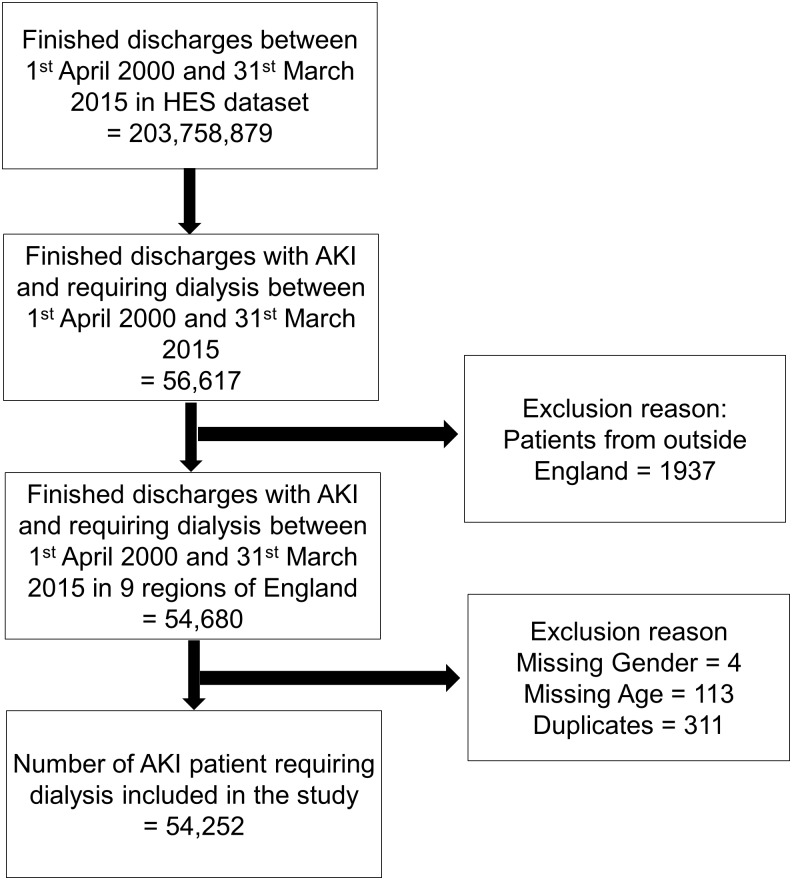
Study flowchart.

There were 203,758,879 completed discharges in England between 1^st^ April 2000 and 31^st^ March 2015. Of these, we identified 54,680 patients who had AKI and required dialysis (Fig 1). After excluding a small number (311 patients were duplicates, 4 patients were missing gender, 113 patients did not have age recorded), we included 54,252 persons with AKI-D for final analysis.

### Variation in demographic characteristics

The demographic characteristics of persons with AKI-D in each region are presented in Tables 1 and 2. The mean age and CCI increased in all regions over the three five-year periods with the South West having the oldest population from 2000 to 2010 and the South East in 2010 to 2015. London had the lowest proportion of patients with white ethnicity and highest proportion of Black and Asian ethnicity as compared to the other eight regions. The proportion of AKI-D events decreased in medical specialities with corresponding increases in other specialities. All regions evidenced an increase in AKI-D during emergency as compared to elective admissions. Over the three five-year periods, there was increase in proportion of persons with AKI-D who had AKI coded in diagnoses codes 3 to 20 in all regions.

**Table 1:** Regional demographic characteristics of persons with AKI-D in England over three-five year period between 2000 and 2015.

				Ethnicity n (%)				Gender n (%)	Admission method n (%)		
		Age^§^	CCI^§^	White	Asian	Black	Not known	Male	Elective	Emergency	Transfer
**2000 to 2005**	London	60·4 (59·1, 61·8)	1 (0·9, 1·1)	289 (44·3)	44 (6·7)	73 (11·2)	212 (32·5)	391 (60)	94 (14·4)	419 (64·3)	136 (20·9)
	North East	62·6 (60·6, 64·5)	1·5 (1·3, 1·6)	247 (76·5)	4 (1·2)	1 (0·3)	70 (21·7)	185 (57·3)	29 (9)	227 (70·3)	66 (20·4)
	North West	60·5 (58·9, 62·1)	1·4 (1·3, 1·6)	279 (59·4)	4 (0·9)	1 (0·2)	182 (38·7)	284 (60·4)	70 (14·9)	240 (51·1)	156 (33·2)
	Yorkshire	64·4 (62·5, 66·2)	1·9 (1·7, 2·1)	179 (65·8)	6 (2·2)	3 (1·1)	76 (27·9)	179 (65·8)	28 (10·3)	126 (46·3)	117 (43)
	East Midlands	61·7 (59·8, 63·5)	1·6 (1·5, 1·8)	303 (75·6)	17 (4·2)	4 (1)	74 (18·5)	252 (62·8)	32 (8)	221 (55·1)	145 (36·2)
	West Midlands	64·8 (63·7, 65·9)	1·2 (1·1, 1·3)	670 (78·3)	70 (8·2)	15 (1·8)	98 (11·4)	560 (65·4)	126 (14·7)	599 (70)	130 (15·2)
	East of England	64·1 (62·5, 65·6)	1·4 (1·2, 1·5)	327 (68·4)	2 (0·4)	1 (1)	143 (29·9)	289 (60·5)	49 (10·3)	340 (71·1)	89 (18·6)
	South East	64·2 (62·9, 65·4)	1·3 (1·2, 1·4)	442 (58·6)	8 (1·1)	4 (0·5)	295 (39·1)	472 (62·6)	89 (11·8)	513 (68)	151 (20)
	South West	65·8 (64·7, 67)	2·2 (2, 2·3)	577 (76·5)	4 (0·5)	2 (0·3)	166 (22)	457 (60·6)	57 (7·6)	567 (75·2)	130 (17·2)
**2005 to 2010**	London	63 (62·3, 63·6)	2·3 (2·2, 2·3)	1287 (55·9)	332 (14·4)	280 (12·2)	240 (10·4)	1403 (60·9)	209 (9·1)	1715 (74·5)	372 (16·2)
	North East	64·1 (63·1, 65)	2·2 (2·1, 2·3)	944 (90·7)	16 (1·5)	3 (0·3)	72 (6·9)	646 (62·1)	105 (10·1)	718 (69)	216 (20·7)
	North West	62·9 (62·3, 63·5)	2·1 (2, 2·1)	1942 (77)	66 (2·6)	22 (0·9)	475 (18·8)	1424 (56·5)	861 (34·1)	1324 (52·5)	334 (13·2)
	Yorkshire	64 (63, 65)	2·1 (2, 2·2)	934 (80·2)	47 (4)	9 (0·8)	161 (13·8)	715 (61·4)	117 (10·1)	742 (63·7)	305 (26·2)
	East Midlands	64·8 (63·9, 65·6)	2·2 (2·1, 2·3)	1213 (87)	62 (4·4)	20 (1·4)	72 (5·2)	880 (63·1)	150 (10·8)	875 (62·8)	366 (26·3)
	West Midlands	64·7 (63·9, 65·4)	2 (1·9, 2·1)	1505 (82·4)	125 (6·8)	53 (2·9)	129 (7·1)	1137 (62·2)	223 (12·2)	1368 (74·9)	232 (12·7)
	East of England	65·1 (64·2, 66)	2 (1·9, 2·1)	989 (77·4)	26 (2)	22 (1·7)	228 (17·8)	779 (61)	158 (12·4)	969 (75·8)	151 (11·8)
	South East	65·9 (65·2, 66·6)	2·1 (2, 2·1)	1646 (79·6)	56 (2·7)	28 (1·4)	311 (15)	1320 (63·8)	221 (10·7)	1564 (75·6)	283 (13·7)
	South West	66 (65·2, 66·8)	2·2 (2·1, 2·3)	1323 (84·3)	10 (0·6)	9 (0·6)	226 (14·4)	995 (63·4)	212 (13·5)	1168 (74·4)	180 (11·5)
**2010 to 2015**	London	64·5 (64·1, 64·9)	2·6 (2·6, 2·6)	3443 (55·6)	994 (16)	727 (11·7)	661 (10·7)	3780 (61)	509 (8·2)	4955 (80)	637 (10·3)
	North East	65 (64·3, 65·7)	2·3 (2·2, 2·3)	1744 (90·3)	36 (1·9)	4 (0·2)	136 (7)	1172 (60·7)	276 (14·3)	1292 (66·9)	250 (12·9)
	North West	64·4 (63·9, 64·8)	2·4 (2·3, 2·5)	3947 (87·3)	147 (3·3)	39 (0·9)	334 (7·4)	2857 (63·2)	614 (13·6)	3331 (73·7)	477 (10·5)
	Yorkshire	64 (63·3, 64·6)	2·3 (2·2, 2·3)	2411 (84·5)	136 (4·8)	22 (0·8)	256 (9)	1730 (60·6)	342 (12)	2101 (73·6)	327 (11·5)
	East Midlands	65·2 (64·6, 65·8)	2·4 (2·4, 2·5)	2454 (85·8)	162 (5·7)	31 (1·1)	147 (5·1)	1721 (60·6)	292 (10·2)	1916 (67)	376 (13·2)
	West Midlands	65·3 (64·7, 65·8)	2·5 (2·4, 2·6)	3001 (80·8)	326 (8·8)	125 (3·4)	199 (5·4)	2317 (62·4)	313 (8·4)	2910 (78·4)	467 (12·6)
	East of England	66·1 (65·6, 66·7)	2·4 (2·3, 2·4)	2701 (82·4)	119 (3·6)	50 (1·5)	363 (11·1)	2021 (61·7)	398 (12·1)	2475 (75·5)	308 (9·4)
	South East	66·2 (65·8, 66·6)	2·4 (2·4, 2·5)	4772 (84·1)	170 (3)	51 (0·9)	586 (10·3)	3580 (63·1)	552 (9·7)	4221 (74·4)	728 (12·8)
	South West	64·5 (63·9, 65·2)	2·4 (2·3, 2·4)	2698 (87·1)	21 (0·7)	14 (0·5)	343 (11·1)	2046 (66)	528 (17)	2148 (69·3)	350 (11·3)

^§^ Mean with 95% Confidence intervals

**Table 2:** Regional admission details of persons with AKI-D in England over three-five year period between 2000 and 2015.

		AKI in diagnosis code n (%)			Main speciality n (%)				
		Primary	Secondary	Other codes	Medicine	Surgery	Intensive care	Cardiothoracic	Died n (%)
**2000 to 2005**	London	436 (66·9)	62 (9·5)	154 (23·6)	591 (90·6)	41 (6·3)	6 (0·9)	13 (2)	168 (25·8)
	North East	154 (47·7)	65 (20·1)	104 (32·2)	250 (77·4)	43 (13·3)	21 (6·5)	9 (2·8)	128 (39·6)
	North West	265 (56·4)	77 (16·4)	128 (27·2)	377 (80·2)	27 (5·7)	61 (13)	5 (1·1)	156 (33·2)
	Yorkshire	178 (65·4)	36 (13·2)	58 (21·3)	234 (86)	22 (8·1)	1 (0·4)	15 (5·5)	73 (26·8)
	East Midlands	249 (62·1)	54 (13·5)	98 (24·4)	342 (85·3)	46 (11·5)	10 (2·5)	3 (0·7)	143 (35·7)
	West Midlands	502 (58·6)	150 (17·5)	204 (23·8)	722 (84·3)	113 (13·2)	2 (0·2)	19 (2·2)	311 (36·3)
	East of England	277 (57·9)	91 (19)	110 (23)	430 (90)	36 (7·5)	6 (1·3)	6 (1·3)	110 (23)
	South East	390 (51·7)	132 (17·5)	232 (30·8)	620 (82·2)	81 (10·7)	34 (4·5)	18 (2·4)	240 (31·8)
	South West	544 (72·1)	86 (11·4)	124 (16·4)	716 (95)	21 (2·8)	12 (1·6)	4 (0·5)	188 (24·9)
**2005 to 2010**	London	955 (41·5)	336 (14·6)	1011 (43·9)	1740 (75·6)	296 (12·9)	180 (7·8)	77 (3·3)	899 (39·1)
	North East	429 (41·2)	162 (15·6)	450 (43·2)	682 (65·5)	160 (15·4)	131 (12·6)	66 (6·3)	464 (44·6)
	North West	1240 (49·2)	357 (14·2)	925 (36·7)	1886 (74·8)	227 (9)	276 (10·9)	131 (5·2)	798 (31·6)
	Yorkshire	603 (51·8)	181 (15·5)	380 (32·6)	948 (81·4)	122 (10·5)	27 (2·3)	66 (5·7)	407 (35)
	East Midlands	749 (53·7)	194 (13·9)	451 (32·4)	1145 (82·1)	140 (10)	52 (3·7)	57 (4·1)	425 (30·5)
	West Midlands	954 (52·2)	271 (14·8)	602 (33)	1521 (83·3)	192 (10·5)	52 (2·8)	57 (3·1)	621 (34)
	East of England	489 (38·3)	221 (17·3)	568 (44·4)	935 (73·2)	172 (13·5)	130 (10·2)	41 (3·2)	434 934)
	South East	861 (41·6)	353 (17·1)	855 (41·3)	1474 (71·2)	342 (16·5)	193 (9·3)	57 (2·8)	764 (36·9)
	South West	893 (56·9)	186 (11·9)	490 (31·2)	1269 (80·9)	108 (6·9)	112 (7·1)	79 (5)	431 (27·5)
**2010 to 2015**	London	1938 (31·3)	880 (14·2)	3378 (54·5)	4572 (73·8)	745 (12)	637 (10·3)	221 (3·6)	2823 (45·6)
	North East	596 (30·9)	291 (15·1)	1044 (54·1)	1054 (54·6)	288 (14·9)	390 (20·2)	194 (10)	905 (46·9)
	North West	1266 (28)	761 (16·8)	2495 (55·2)	2555 (56·5)	638 (14·1)	827 (18·3)	489 (10·8)	2264 (50·1)
	Yorkshire	899 (31·5)	491 (17·2)	1463 (51·3)	1991 (69·8)	573 (20·1)	63 (2·2)	220 (7·7)	1267 (44·4)
	East Midlands	1016 (35·5)	397 (13·9)	1446 (50·6)	1905 (66·6)	389 (13·6)	297 (10·4)	261 (9·1)	1196 (41·8)
	West Midlands	1338 (36)	654 (17·6)	1722 (46·4)	2792 (75·2)	433 (11·7)	294 (7·9)	190 (5·1)	1514 (40·8)
	East of England	942 (28·7)	476 (14·5)	1859 (56·7)	2051 (62·6)	488 (14·9)	494 (15·1)	240 (7·3)	1447 (44·2)
	South East	1893 (33·4)	859 (15·1)	2924 (51·5)	3858 (68)	903 (15·9)	579 (10·2)	323 (5·7)	2364 (41·6)
	South West	1227 (39·6)	392 (12·7)	1479 (47·7)	2165 (69·9)	273 (8·8)	328 (10·6)	332 (10·7)	1066 (34·4)

### Regional variation in population incidence of AKI-D

**Fig 2:**
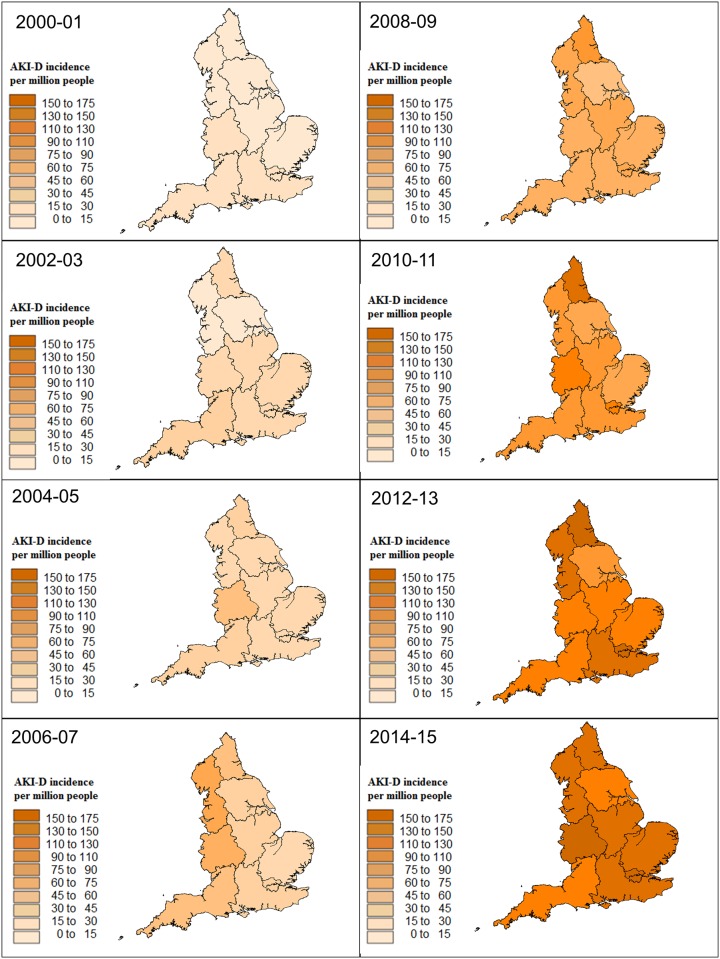
Regional variation in population incidence of AKI-D in England between 2000 and 2015.

In the period 2000–01, the population incidence of AKI-D was lowest in Yorkshire at 7·9 per million people (pmp) and highest in the West Midlands at 25·6 pmp with a regional variation, defined as the ratio of maximum to minimum incidence, of 3·3 fold (Fig 2). Over 15 years, the number of cases and population incidence of AKI-D increased in all regions. In 2014–15, the population incidence was highest in London at 157·8 pmp and lowest in the South West at 119·5 pmp. The regional variation decreased to 1·3 fold (p<0·001 versus 2000–1). During the study period from 2000–01 to 2014–15, Yorkshire had the greatest increase in incidence of AKI-D (16·5 fold) as compared to the South West (5·1 fold), which had the smallest increase. From 2011–15, London continued to have highest incidence of AKI-D reaching a peak in 2012–13 at 166·6 pmp.

### Regional variation in incidence of AKI-D expressed per hospitalization

**Fig 3:**
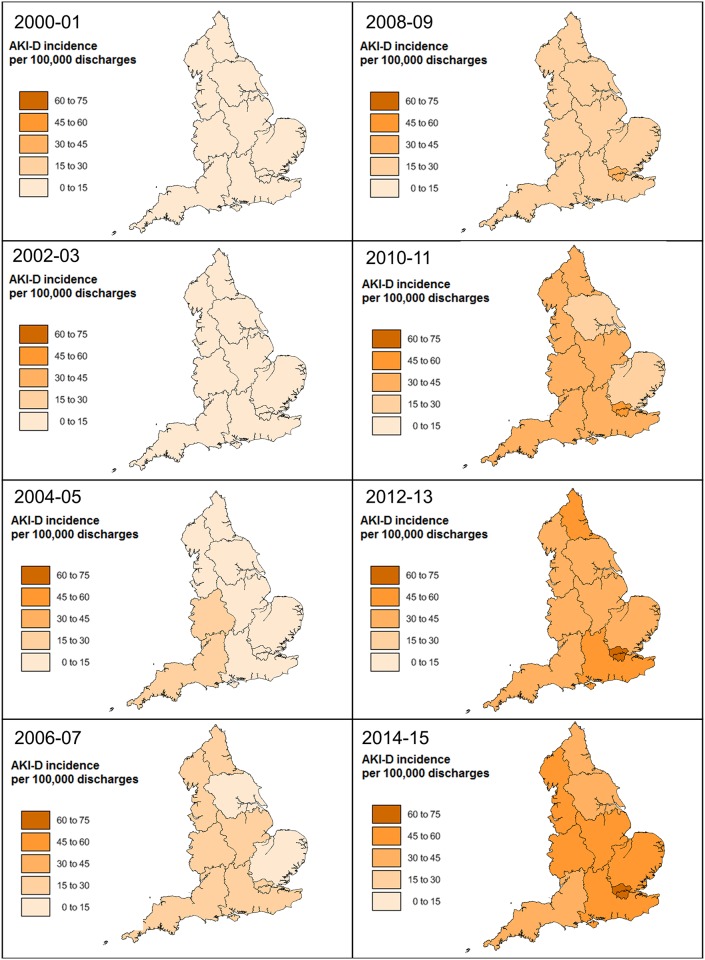
Regional Incidence of AKI-D per 100,000 hospital discharges in England between 2000 and 2015.

Yorkshire had the lowest incidence of AKI-D when expressed per 100,000 hospital discharges amongst all regions for 13 years of the 15-year study period (Fig 3). In the year 2000–01, the incidence of AKI-D was highest in the West Midlands at 11·6 per 100,000 hospital discharges and lowest in Yorkshire at 3·2 cases per 100,000 hospital discharges, with a regional variation of 3·6 fold. This regional variation decreased to 1·6 fold in 2014–15. Yorkshire had a 12·6-fold increase in AKI-D over the 15-year period from 3·2 to 41·3 cases per 100,000 hospital discharges. The South West had only a 3·9-fold increase in AKI-D from 10·3 to 39·9 cases per 100,000 hospital discharges. From 2008 to 2014–15, London continued to have the highest incidence of AKI-D per 100,000 hospitalizations, reaching a peak in 2012–13 at 70 cases per 100,000 hospital discharges.

### Regional variation in unadjusted case-fatality in AKI requiring dialysis

**Fig 4:**
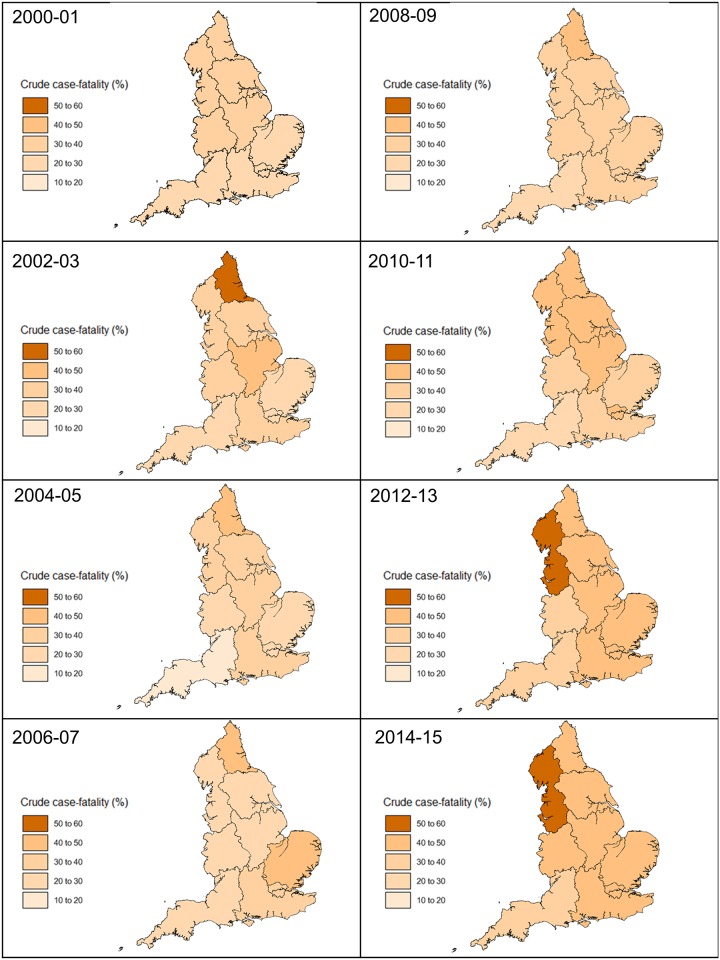
Regional case-fatality in persons with AKI-D in England between 2000 and 2015.

The unadjusted case-fatality was lowest in the East of England (23·7%) and highest in the West Midlands (37·8%) in 2000–01 (Fig 4). The South West had the lowest unadjusted case-fatality from 2008–09 to 2014–15, while the North West had the highest unadjusted case-fatality from 2010–11 to 2014–15, reaching 52·8% in the later year. Unadjusted case-fatality increased in all regions over 15 years, with London having the greatest increase from 26·1% to 46·1%. The lowest increase was in the West Midlands, from 37·8% to 45·2%. In the last five-year period, North West had highest unadjusted case-fatality.

### Multivariable adjusted case-fatality

**Fig 5:**
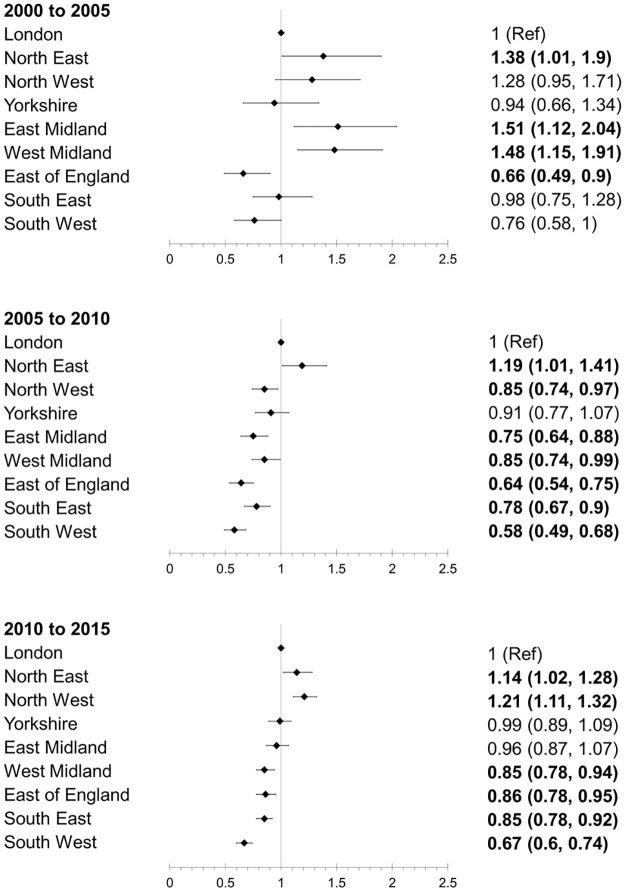
Multivariable adjusted case-fatality over 15 years in each region over three 5-year periods with London as reference region.

Using London as the reference, in the period of 2000–2005, the North East (OR 1·38; 95%CI 1·01, 1·90), East Midlands (OR 1·38; 95%CI 1·01, 1·90) and West Midlands (OR 1·38; 95%CI 1·01, 1·90) had higher odds for death, while East of England had significantly lower odds for death (OR 0·66; 95% CI 0·49, 0·90) (Fig 5). The North East had higher odds for death in all three 5-year periods. In 2005–10 and 2010–15, the West Midlands, East of England, South East and South West had lower odds for death as compared to London.

### Adjusted case fatality in each region over 15 years

**Fig 6:**
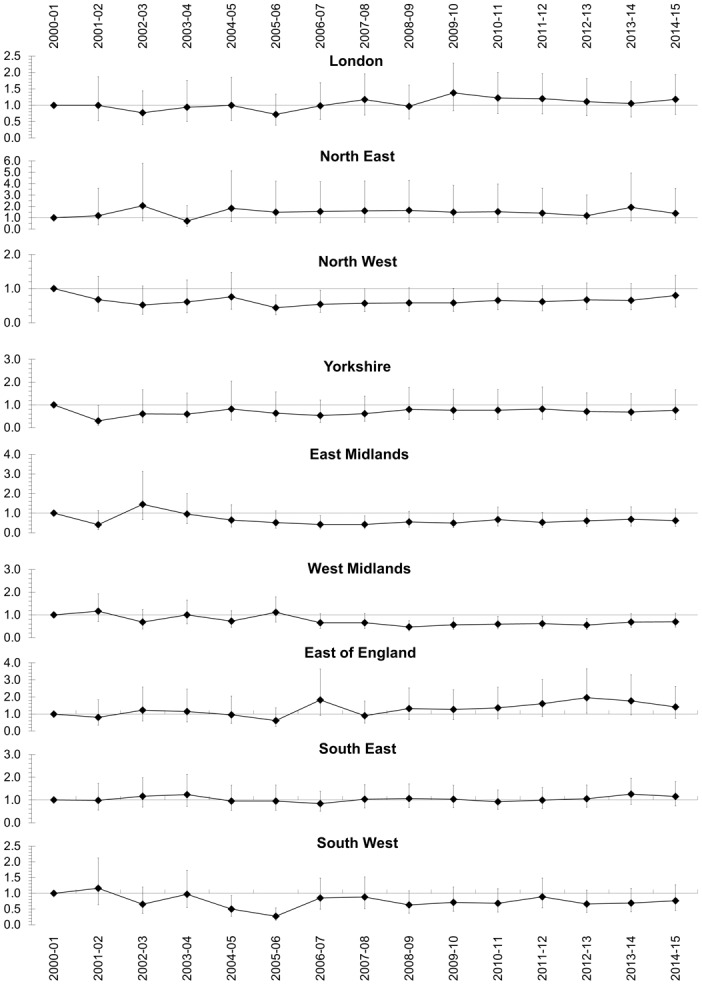
Multivariable adjusted case-fatality in each region of England over 15 years with 2000–01 as reference period.

As compared to 2000–01, the multivariable adjusted case-fatality did not improve in any of the 15 years in London, the North East, Yorkshire, East of England and the South East (Fig 6). The adjusted odds for death were lower in the West Midlands from 2008–09 to 2012–13 as compared to 2000–01. However, there was no statistical difference in odds for death in all regions in 2014–15 as compared to 2000–01.

Our findings, in sensitivity analyses that included all cases with “ethnicity unknown” (S1 Fig) or that included CCI as continuous variables (S2 Fig), remained unchanged.

### Nephrology workforce

The density of nephrology workforce increased in all regions during the first 12-years of the study period after which most regions showed a decline in nephrology workforce (S1 Table). The East Midlands had the lowest density of nephrologist per 100,000 people from 2000 to 2008. London had the highest number of nephrologist per 100,000 people in each of the periods studied, reaching highest levels in 2014–15 at 6·31 per 100,000 people, while the South East had the lowest density of Nephrologists in 2014–15 at 1·86 per 100,000 people. There was a positive correlation between the nephrology workforce density and population incidence of AKI-D in all regions, the highest being in East Midlands, r = 0·923, p < 0·001.

## Discussion

This study is the first to investigate regional variation over time in the epidemiology of AKI-D in a single healthcare system over a period of 15 years. We found significant regional variation in the population incidence and case-fatality of AKI-D in each year of the study period. The adjusted case-fatality remained high in the North East followed by London in all three five-year periods but decreased in most other regions during the last ten years. However, within the regions, there was no improvement in adjusted case-fatality in 2014–15 as compared to 2000–01.

### Implications of variation in incidence

This study shows that there was considerable variation in the regional population incidence of AKI-D that has decreased over 15 years but remains significant. The incidence of AKI-D per 100,000 hospital discharges showed a similar trend. Some of the regional variation may be accounted for by differences in population demographic characteristics but this in unlikely to explain all of the variation. For example, London had the lowest population age but the highest incidence of AKI-D in 2010–15. Other factors that may have influenced the incidence of AKI-D include access to primary care physicians, effectiveness of strategies to prevent AKI or availability of nephrology services and dialysis. Hsu and colleagues also reported considerable regional variation in the incidence AKI-D in the United States. However, the study did not evaluate temporal changes in regional epidemiology and also lacked detailed geographical granularity as only four regions were analyzed [6]. The study found that the region with most nephrologists had lowest incidence of AKI-D though some of this variation could be attributed to the additional factor of multiple private healthcare providers. They also found that the region with highest incidence of persons with AKI-D had the lowest case-fatality. These findings are in contrast to our study where London had the highest incidence of AKI-D, highest case-fatality and the greatest number of nephrologists per 100,000 population. The importance of identifying variation in the incidence of AKI-D that is not attributable to population demographics is that it implies that incidence could be reduced in some regions by addressing the factors responsible for variation.

### Regional variation in case-fatality

We found considerable variation not only in the incidence of AKI-D, but also case-fatality. While no region showed a sustained improvement in adjusted case-fatality, temporary improvement was seen in some regions and the relative case fatality in regions changed over time. Yorkshire had the lowest incidence of AKI-D per hospitalization but the highest unadjusted case-fatality in the last five years. In this analysis we were able to adjust for important patient characteristics and the observed variation in case-fatality was therefore independent of these. Several factors may contribute to variation in the case-fatality associated with AKI-D including variation in the timeliness of diagnosis and intervention, variation in distribution of nephrology departments that provide acute dialysis and variable delay in transfer to dialysis centers [16]. The North East and North West regions had a significantly higher proportion of persons with AKI-D in intensive care and this may indicate either increased severity of illness or lack of acute dialysis in nephrology units. Timing of initiation of dialysis could be another factor though there is no agreed threshold for commencing dialysis in AKI and this has been subject to many studies in critically ill patients [17, 18]. Though some studies have reported encouraging reduction in variation, this is not uniform for many other diseases [19–21]. In this study, the decrease in the variation in incidence of AKI-D is encouraging, but the observed variation in case-fatality independent of patient characteristics suggests that survival could be improved by addressing factors responsible for the variation. This is particularly important, because the lack of specific treatments for AKI implies that the best option to improve outcomes at present is to improve the quality and timeliness of supportive care

### Strengths and limitations of the study

This study has several notable strengths. Coming from a universal healthcare system, this analysis provides significant insight in the regional epidemiology of AKI-D and unlike studies in other health care systems, is unlikely to have been influenced to the same extent by capitation or variation between different healthcare providers. This study interrogated the HES dataset, which consists of routinely available data that is used for investment, activity and monitoring outcomes of the whole population in need and not just those who make contact with a particular service. Several limitations should be considered when interpreting these findings. First, we were unable to assess the degree to which regional variations were influenced by physician preference or procedural appropriateness. Second, these results were not linked to specific healthcare centers and we were therefore unable to assess the impact of availability of acute dialysis services. Third, we were unable to adjust for unknown confounders, which may influence analysis in any observational study. This is particularly true for the population incidence.

## Conclusions

Within a single national health service**,** we have observed considerable regional variation in the incidence and case fatality associated with AKI-D that was not entirely attributable to variations in demographic or other identifiable clinical factors. The extent of regional variation has decreased over the past 15 years but remains significant. This robust observation makes a compelling case for further research to elucidate the reasons for the variation to identify modifiable factors and interventions that may reduce the incidence and case fatality in all regions to levels observed in the best-performing regions.

## Supporting Information

S1 Fig Multivariable adjusted case-fatality over 15 years in each region over three 5-year periods with London as reference region after excluding unknown ethnicity. (TIF)

S2 Fig Multivariable adjusted case-fatality over 15 years in each region over three 5-year periods with London as reference region and with CCI as continuous variable. (TIF)

S1 STROBE Checklist (DOC)

S1 Table Supplementary appendix. Regional nephrology workforce in England between 2000 and 2015. Sensitivity analysis. (DOC)
