# Supplementary figures and images for: Regional Variation in Acute Kidney Injury Requiring Dialysis in the English National Health Service from 2000 to 2015 – A National Epidemiological Study
Source: PLoS One. 2016 Oct 17;11(10):e0162856. doi: 10.1371/journal.pone.0162856 (PMC5066970; doi:10.1371/journal.pone.0162856)

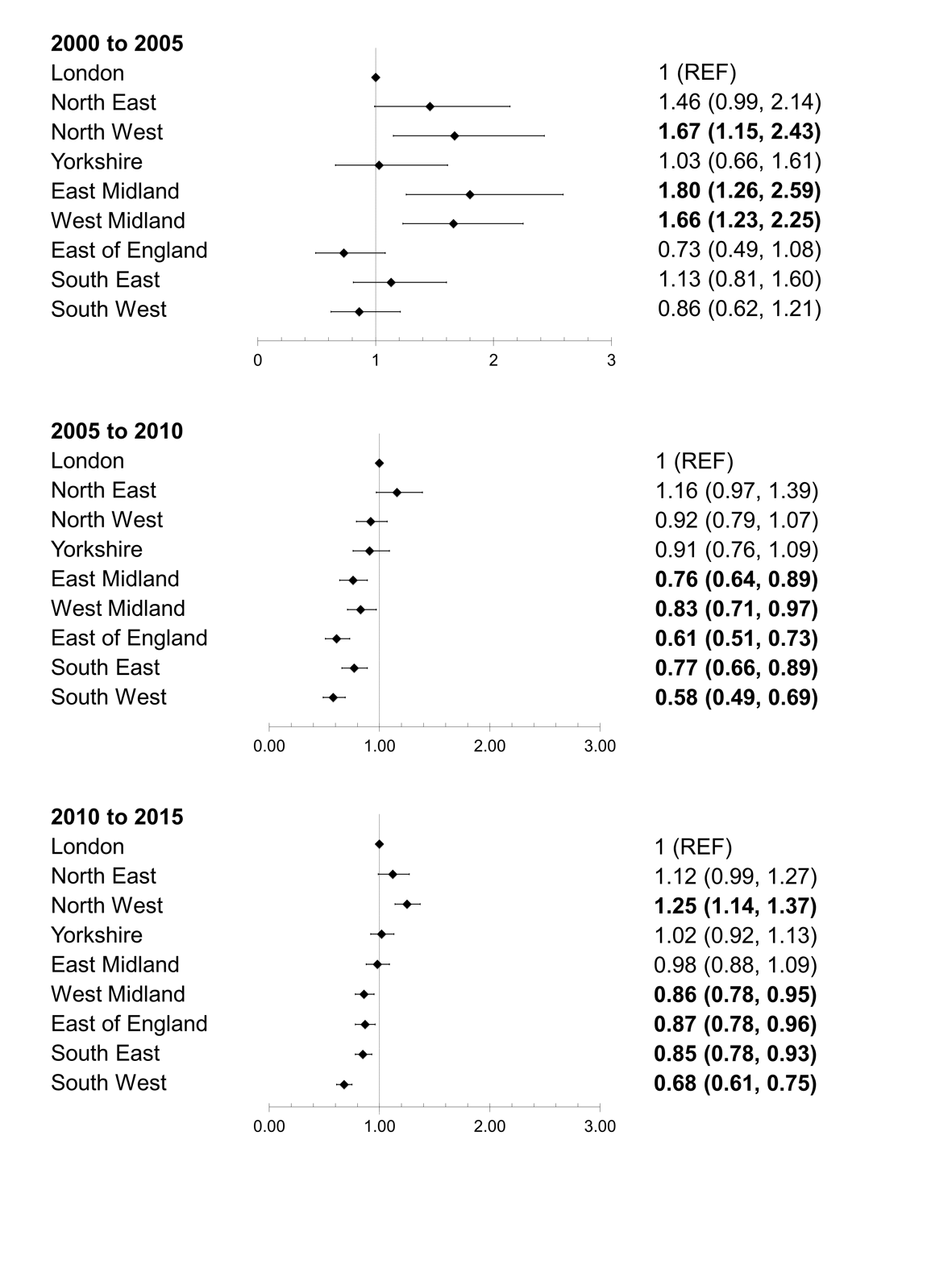

Supplement: S1 Fig — (TIF) [file pone.0162856.s001.tif]

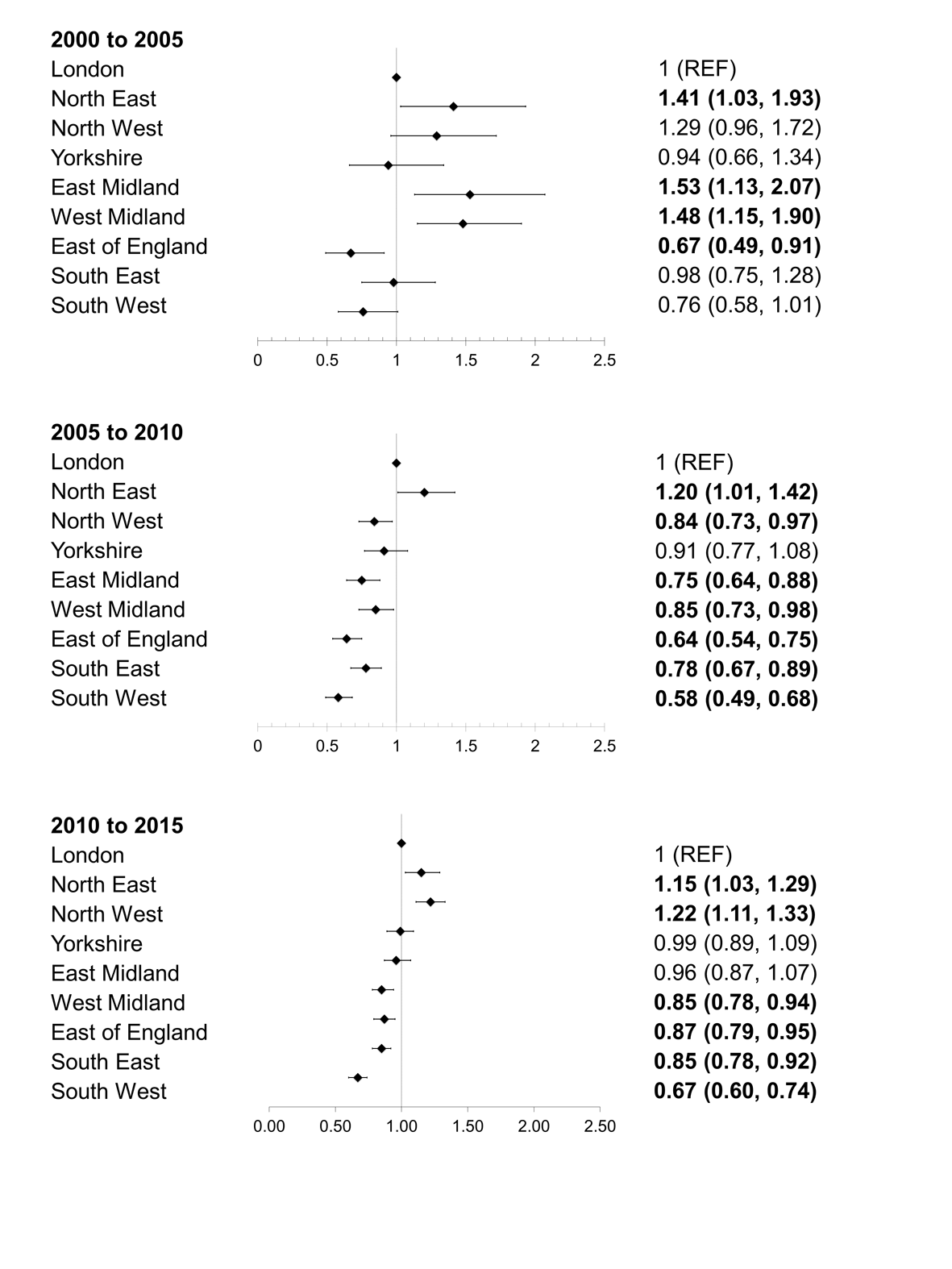

Supplement: S2 Fig — (TIF) [file pone.0162856.s002.tif]
