# Supplementary material for: Regional Variation in Acute Kidney Injury Requiring Dialysis in the English National Health Service from 2000 to 2015 – A National Epidemiological Study
Source: PLoS One. 2016 Oct 17;11(10):e0162856. doi: 10.1371/journal.pone.0162856 (PMC5066970; doi:10.1371/journal.pone.0162856)
Supplement: S1 Table — Regional nephrology workforce in England between 2000 and 2015. Sensitivity analysis. (DOC) [file pone.0162856.s004.doc]

**Supplementary appendix**

**Regional variation in acute kidney injury requiring dialysis in English National Health Service from 2000 to 2015 – a national epidemiological study.**

Nephrology workforce between 2000 and 2015 in England

We obtained number of qualified nephrologists in each region from 2000 to 2015 from Health and Social Care Information Centre (HSCIC) published in the annual census of medical and dental staff in the NHS.

S1 table: Nephrology workforce density per 100,000 people in England from 2000-2015

| Year | North East | North West | Yorkshire | East Midlands | West Midlands | East of England | London | South East | South West |
| --- | --- | --- | --- | --- | --- | --- | --- | --- | --- |
| 2000-01 | 1.59 | 1.45 | 1.58 | 1.06 | 2.35 | 1.83 | 4.01 | 1.51 | 1.61 |
| 2001-02 | 1.92 | 1.59 | 1.88 | 1.25 | 2.52 | 1.90 | 4.87 | 1.58 | 1.76 |
| 2002-03 | 1.68 | 1.82 | 1.93 | 1.38 | 2.85 | 2.10 | 4.70 | 1.85 | 2.06 |
| 2003-04 | 1.63 | 1.81 | 2.22 | 1.61 | 3.43 | 2.14 | 4.73 | 1.89 | 2.08 |
| 2004-05 | 1.78 | 1.91 | 2.39 | 1.64 | 3.45 | 2.32 | 5.45 | 1.83 | 2.13 |
| 2005-06 | 1.77 | 2.14 | 2.09 | 1.70 | 3.41 | 2.42 | 4.73 | 1.76 | 2.37 |
| 2006-07 | 1.67 | 2.13 | 2.18 | 1.55 | 2.94 | 2.63 | 5.13 | 1.81 | 1.99 |
| 2007-08 | 1.97 | 2.20 | 2.01 | 1.52 | 3.34 | 2.63 | 5.27 | 1.64 | 1.76 |
| 2008-09 | 1.97 | 2.16 | 2.39 | 1.78 | 4.05 | 2.95 | 4.88 | 1.75 | 1.91 |
| 2009-10 | 1.99 | 2.38 | 2.51 | 1.85 | 3.68 | 2.62 | 5.54 | 2.29 | 2.26 |
| 2010-11 | 1.75 | 2.58 | 2.34 | 2.27 | 3.92 | 2.65 | 5.02 | 1.94 | 2.50 |
| 2011-12 | 1.71 | 2.78 | 2.42 | 2.42 | 3.41 | 2.69 | 5.05 | 2.11 | 2.43 |
| 2012-13 | 1.95 | 2.73 | 2.50 | 2.30 | 3.65 | 2.83 | 5.62 | 2.39 | 2.39 |
| 2013-14 | 1.89 | 2.50 | 2.37 | 2.38 | 3.40 | 2.69 | 6.26 | 1.83 | 2.16 |
| 2014-15 | 2.04 | 2.48 | 2.44 | 2.45 | 3.38 | 2.53 | 6.31 | 1.86 | 2.22 |

Sensitivity analysis for dialysis requiring AKI

Two sensitivity analyses were performed: First, we determined whether there was a difference in the effect size with improvement in ethnicity recording in HES. Sensitivity analysis was performed to ascertain the effect of “unknown ethnicity” by excluding all cases where ethnicity was recorded as “unknown” (S1 Fig). Second, we performed sensitivity analysis to ascertain the effect of Charlson’s comorbidity index as continuous variables (S2 Fig). The associations between discharge status (dead or alive) and gender, age group, period of discharge, AKI in diagnoses codes, method of admission, Charlson’s co-morbidity index, ethnicity and regions were analyzed using multi variable logistic regression. We used London as reference to evaluate improvement in case-fatality in other regions. The results of both sensitivity analyses were essentially similar to the primary analysis (Fig 5 in main manuscript).
